# ^222^Rn and ^226^Ra activity concentrations in groundwaters of southern Poland: new data and selected genetic relations

**DOI:** 10.1007/s10967-014-3215-x

**Published:** 2014-06-08

**Authors:** Tadeusz A. Przylibski, Joanna Gorecka, Agata Kula, Lidia Fijałkowska-Lichwa, Katarzyna Zagożdżon, Paweł Zagożdżon, Wojciech Miśta, Robert Nowakowski

**Affiliations:** 1 Division of Geology and Mineral Waters, Institute of Mining Engineering, Faculty of Geoengineering, Mining and Geology, Wrocław University of Technology, Wybrzeże S. Wyspiańskiego 27, 50-370 Wrocław, Poland; 2 Faculty of Geoengineering, Mining and Geology, Graduated Master of Science of Wrocław University of Technology, Wybrzeże S. Wyspiańskiego 27, 50-370 Wrocław, Poland

**Keywords:** Radon, Radium, Groundwater, Radon water, Liquid-scintillation spectrometry

## Abstract

Since 2008, the authors have been conducting research into ^222^Rn and ^226^Ra activity concentrations in shallow circulation groundwaters in southern Poland. Measurements have been performed with a liquid-scintillation method and ultra low-level liquid-scintillation spectrometers α/β Quantulus 1220. The research carried out so far has demonstrated that in the Sudetes groundwaters with high activity concentrations of ^222^Rn and ^226^Ra are common. In other studied areas in southern Poland no shallow circulation groundwaters with high radon or radium concentrations have been found yet. The conducted research has demonstrated that the activity concentration of ^222^Rn dissolved in shallow circulation groundwaters in the Sudetes depends chiefly on the amount of radon, which after being released as gas from reservoir rocks is dissolved in waters flowing through these rocks. At the same time, the concentration of ^222^Rn dissolved in some shallow circulation groundwaters in the Carpathians is influenced significantly by the amount of radon produced from the decay of its parent ion ^226^Ra^2+^ dissolved in these waters.

## Introduction

So far, data on radon occurrence in groundwaters in Poland have been available mostly for the Sudetes and they have related only to the longest-lived isotope ^222^Rn [1–13]. Rarely results of radon research in groundwaters of other regions of Poland have been published [14, 15]. Far more rarely, data on radium content in Polish groundwaters have been published. They usually concerned isotopes ^226^Ra and ^228^Ra and mostly also referred to the Sudetes [10, 13, 16] and only rarely to other areas, particular towns, villages or groundwater intake complexes outside this mountain range [17–21]. This is due to the fact that in Poland it is only in the Sudetes where rocks containing increased or even deposit concentrations of uranium lie shallow under the ground or directly on its surface [7, 22].

All the mentioned isotopes, i.e. ^222^Rn, ^226^Ra and ^228^Ra, play the key role when it comes to radioactive properties of groundwaters [23]. Because of this, any information concerning concentrations of these radionuclides in groundwater is important from the radiological point of view. It is also important for protecting people from the increased exposure to ionizing radiation from waters intended for human consumption in those areas where groundwaters can be rich in the radon and radium isotopes in question. A much bigger health hazard is linked to radium isotopes, as radon can be easily removed from water by its intensive aeration before it is fed into the water supply system. Additionally, waters containing large amounts of dissolved ^222^Rn can be, and in many countries are, regarded as medicinal and used in balneotherapy (e.g. [2–4, 7, 9, 24]).

For many years, the authors have been measuring the activity concentrations of ^222^Rn, and recently also of ^226^Ra, in Polish groundwaters. These results can now be published thanks to obtaining new data on the occurrence of ^222^Rn and ^226^Ra in shallow circulation groundwaters in southern Poland. This will enable supplementing information about the occurrence of these radionuclides, very important in terms of natural radioactivity of groundwaters and determining certain genetic relationships between these isotopes in the studied groundwaters in southern Poland. The discussed results were obtained from research which is still in progress and they sum up one of its successive stages. This will enable specifying the subsequent aims of further research and problems to be solved.

## Research area

The research was carried out in southern Poland, the area comprising large geological units such as the Sudety Mountains, the Carpathian Mountains and rocks of the Mesozoic platform cover in the border zone between the West European and the East European Platforms (Teisseyre-Tornquist zone). Owing to the location of the authors’ research institute, the largest amount of data was collected from the Sudetes, and much less—from the Carpathians and the Mesozoic cover in the Teisseyre-Tornquist zone.

The main area of the authors’ research is the Sudetes. They are the NE part of the Bohemian Massif—the largest crystalline massif of Central Europe. They are built of rocks folded and uplifted during the Variscan orogeny. Their relief was rejuvenated by younger tectonic movements of the Alpine orogeny, which caused the uplifting of today’s Sudetic ranges along numerous faults forming horsts and grabens. Because of this, the present-day Sudetes are a geological mosaic with a large variety of crystalline rocks occurring in a small area—on the surface or under just a few metres of rock waste. Igneous rocks (like granitoids or ryolites) and metamorphic rocks (gneisses and many varieties of schists) can be found in close proximity to each other. The latter are very often rich in uranium [7, 22, 25–29].

The Carpathians are an Alpine orogen forming an arc of over 1,300 km in length, bent to the NE, stretching from the Vienna Forest in the west to the Iron Gate of the Danube River at the Serbian–Romanian border in the east. In the west, the Carpathians are linked with the eastern Alps, and in the east they pass into the Balkan chain. The pieniny klippen belt (PKB), a Neogene suture zone, marks the border between the Outer Carpathians, built chiefly of flysch series composed of sandstones, mudstones and conglomerates formed between the Upper Jurassic and the Lower Miocene, and the older deposits of the Inner Carpathians. The Inner Carpathians are split into numerous horst blocks (so-called core mountains) separated by small Late Tertiary intramontane basins and embayments of the Pannonian Basin. There are two principal tectonic units within the Inner Carpathians: thick-skinned thrust sheets comprising the pre-Alpine crystalline basement along with its Late Palaeozoic-Mesozoic sedimentary cover, and detached cover nappe systems containing Late Palaeozoic to Mesozoic sedimentary rocks with rare volcanics [30–32].

The authors have also taken a few samples from karst groundwater springs flowing out of carbonate rocks (limestones, marls, opokas and chalk) deposited in the Cretaceous. They belong to a Mesozoic sedimentary complex covering older Pre-Cambrian and Palaeozoic rocks, which form the basement of the West European and East European Platforms in the Teisseyre-Tornquist zone (Trans-European Suture Zone). The rocks covering both platforms were deposited in the sea basins of the Mid-Polish Trough chiefly in the Jurassic and the Cretaceous. In the Cainozoic, they were only covered by a thin sheet of clastic rocks. In the regions of Roztocze and Lublin Upland, Cretaceous rocks form extensive outcrops with varied relief, as between the Late Cretaceous and the Quaternary they were subject to block uplifting along faults with NW–SE and SW–NE orientations [33–35]. In the areas of morphological margins, karst springs are common.

**Fig. 1:**
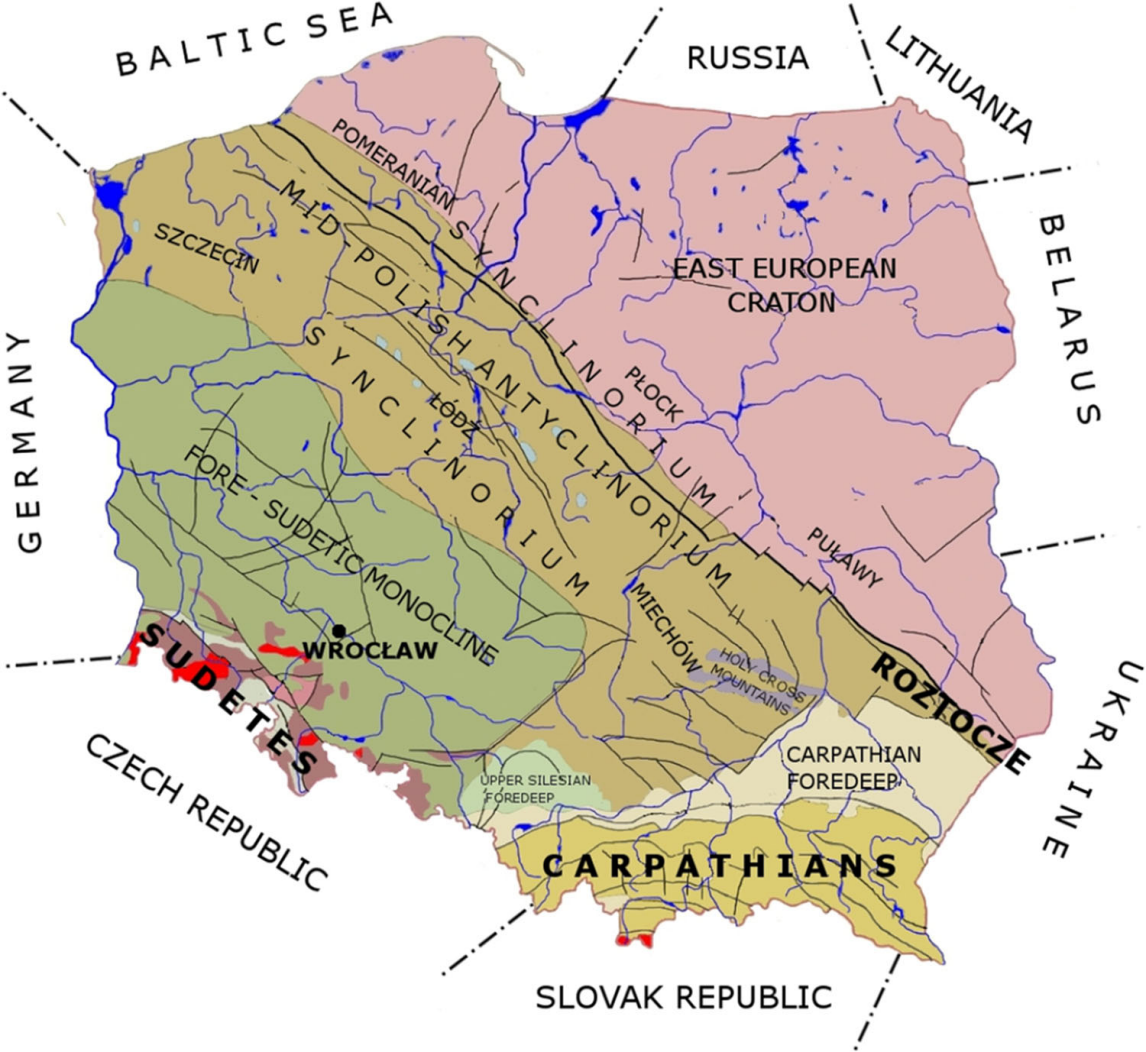
Location of the authors’ research areas, i.e. the Sudetes, the Carpathians and Roztocze, against a simplified geological map of Poland

All the briefly characterized research areas are shown in bold type font in Fig. 1.

## Measurement method

The research focused chiefly on shallow circulation groundwaters from the first aquifer. These are usually waters with the highest level of ^222^Rn activity concentration [36]. The authors took samples from springs, bog springs and seeps, as well as from shallow dug wells (with a few to a few dozen meters in depth), and occasionally also from mine adits and boreholes.

^222^Rn and ^226^Ra activity concentrations were measured by the authors themselves in the Hydrogeological Laboratory of the Division of Geology and Mineral Waters, Institute of Mining Engineering, Wrocław University of Technology. They used two ultra-low-level liquid scintillation spectrometers α/β Quantulus 1220 and the laboratory’s own internal procedure ensuring the appropriate quality of the obtained results.

Before starting the field work, the authors filled measuring vessels of 25 cm^3^ with 10 cm^3^ of liquid scintillator Insta Fluor Plus™. The vessels were made of ^40^K-depleted glass and they were closed tightly with a plastic screw cap. In the field, a disposable syringe was used to take an accurately measured volume of 10 cm^3^ of groundwater. After fitting the syringe with a needle with the inside diameter of 0.9 mm or 1.1 mm, the authors filled up the scintillation vessel by transferring 10 cm^3^ of water from the syringe to the bottom of the measuring vessel, under the scintillator layer. Then, after securing the cap tightly to prevent radon escape, they shook the measuring vessel energetically about a dozen times. This enabled ^222^Rn release from the water and its dissolution in the scintillator. It was possible due to radon’s better solubility in the scintillator than in water. After recording the date and the precise time (with 1 min accuracy) of taking the water sample, the vessel was transported to the laboratory.

In the laboratory, at least 4 h after taking the sample, i.e. the time necessary for establishing the radioactive equilibrium between ^222^Rn and its short-lived progeny in the sample, the proper measurement was started. The measurement vessel was placed inside the spectrometer and then nine one-hour long measurements were performed at intervals of several to about a dozen hours. During each measurement, impulses coming from alpha particles formed through the decay of ^222^Rn and its short-lived progeny were counted. The obtained number of impulses from each of the nine measurements was converted to ^222^Rn activity concentration during that measurement. The spectrometer had been previously calibrated with certified calibration standards made of ^226^Ra solutions with known radioactivity levels. The activity concentrations of the ^226^Ra standards used (in Bq/dm^3^) are as follow: 2.2, 5.4, 10.8, 54, 108, 216, 555, 603, 1,111, 1,323, 2,222, 2,645 and 3,638. Each result obtained in this way was converted into ^222^Rn activity concentration at the moment of taking the sample. The researchers used the law of radioactive decay and the known time that had elapsed from the moment of taking the sample to the measurement. In this way, the authors obtained nine values of ^222^Rn activity concentration for each sample. What they accepted as the ultimate result—the value of ^222^Rn activity concentration in the examined groundwater sample at the moment of taking it from the field—was the value of the weighted average of the nine obtained measurement results. The weight was uncertainty of result for each of the nine measurements. In this way, the measurements ensured detection of ^222^Rn activity concentration in water samples from the level of 0.05 Bq/dm^3^, with uncertainty not exceeding 5 %.

The result of ^222^Rn activity concentration obtained in this way could be a bit higher than the real amount of ^222^Rn dissolved in groundwater due to the constant production of ^222^Rn in a water sample from alpha decay of the parent ^226^Ra dissolved in this water. In order to apply a certain correction, the authors measured ^222^Rn activity concentration in the same water sample (the same vessel) and in the same way, after a time necessary for the total decay of the ^222^Rn which was not produced in the sample from the parent ^226^Ra^2+^ dissolved in it. In practice, it was enough for ^222^Rn activity concentration to decrease below the detection limit, i.e. below the value of 0.05 Bq/dm^3^. For most of the measured samples, this time was shorter than 1 month, while for samples of high-radon water (with ^222^Rn activity concentration of 1,000–9,999.9(9) Bq/dm^3^, according to Przylibski’s classification [7]), it could reach even 2–3 months.

The ensuing measurement of ^222^Rn activity concentration, which at that time was already in radioactive equilibrium with parent ^226^Ra^2+^ dissolved in the water sample, provided two practical pieces of information. Firstly, it enabled determining ^226^Ra activity concentration in the water sample, which turned out to be equal to the measured ^222^Rn activity concentration. The values of ^226^Ra activity concentration obtained in this way were the ultimate results of ^226^Ra activity concentration in the studied groundwater samples. These results were also used as input data for calculating statistical parameters characterising ^226^Ra content in the studied groundwaters from southern Poland. These parameters are shown in Table 1. Secondly, knowing the number of impulses measured by the spectrometer and originating from the decay of ^222^Rn being in radioactive equilibrium with ^226^Ra^2+^ dissolved in the water sample, the authors applied an appropriate correction to the initially calculated value of ^222^Rn activity concentration. That value had been obtained from the first ^222^Rn measurement, conducted within a few days after bringing the sample from the field to the laboratory. One should note that only exceptionally, in a few cases, this correction was higher than the uncertainty of ^222^Rn determination, and made it necessary to change the ultimate value of ^222^Rn activity concentration. Precisely, it was necessary to decrease it by the amount of radon that had been produced from the ^226^Ra^2+^ dissolved in the measured groundwater sample during the measurements of ^222^Rn activity concentration. Such cases occurred when ^226^Ra activity concentration was higher than the uncertainty of ^222^Rn activity concentration determination, i.e. c.5 %. This concerned only groundwaters with low ^222^Rn activity concentration i.e. low-radon waters and radon-poor waters (according to Przylibski’s classification [7], having ^222^Rn activity concentration of 10–99.9(9) and 1–9.9(9) Bq/dm^3^ respectively), with ^226^Ra activity concentration higher than 0.1–1.0 Bq/dm^3^.

**Table 1:** Basic statistical parameters describing ^222^Rn and ^226^Ra activity concentrations in shallow circulation groundwaters occurring in the areas of the Sudetes, the Carpathians and Roztocze, as well as all the analysed waters from southern Poland

		Sudetes	Carpathians	Roztocze	Southern Poland
Points of measurement number	^222^Rn ^226^Ra	155 155 (70)	31 31 (4)	7 7 (0)	193 193 (74)
Minimum (Bq/dm^3^)	^222^Rn ^226^Ra	2.03 <0.05 (0.05)	0.20 <0.05 (0.19)	5.10 <0.05	0.20 <0.05 (0.05)
Maximum (Bq/dm^3^)	^222^Rn ^226^Ra	3,043 1.77	104.2 0.62	10.2 <0.05	3,043 1.77
Arithmetic mean (Bq/dm^3^)	^222^Rn ^226^Ra	541.4 (0.19)	16.6 (0.51)	7.9 Nc	437.7 (0.20)
Standard deviation (Bq/dm^3^)	^222^Rn ^226^Ra	480.2 (0.29)	23.2 (0.21)	1.9 Nc	478.6 (0.30)
Median (Bq/dm^3^)	^222^Rn ^226^Ra	444.9 (0.08)	9.7 (0.62)	8.9 Nc	355 0.09
Correlation coefficient		(−0.125)	(0.996)	Nc	(−0.184)

Values in parentheses are related to these measurements, where ^226^Ra activity concentration were above detection limit (>0.05 Bq/dm^3^)—values below 0.05 Bq/dm^3^ are excluded from the calculations of presented statistical parameters

*Nc* not calculated

## Results and discussion

In 2008–2013, the authors performed over 500 measurements of ^226^Ra and ^222^Rn activity concentration in shallow circulation groundwater samples. The measurements were performed in the Hydrogeological Laboratory of the Division of Geology and Mineral Waters, Institute of Mining Engineering, Wrocław University of Technology. The samples had been taken from 193 locations in southern Poland. These 193 locations included 155 ones in the Sudetes, 31—in the Carpathians and 7—in the area of Roztocze. ^222^Rn activity concentration was determined at least once (about a dozen times at the most) at each location, and ^226^Ra activity concentration was determined from one to a few times in waters taken from only 74 of these places. At the other locations, ^226^Ra activity concentration in groundwater was lower than the detection limit for the employed measurement method (<0.05 Bq/dm^3^). The mean values of ^222^Rn and ^226^Ra activity concentration in groundwater from each location were considered for the analysis. Usually there were not more than five measurements at the same spring or well and all values were close one to the other. The results of these analyses, demonstrated by selected basic statistical parameters, are shown in Table 1.

The highest values of activity concentration, both for ^222^Rn and ^226^Ra, were recorded in the Sudetes, while the lowest ones were found in karst groundwaters from Roztocze. While the lowest values, both of ^226^Ra and ^222^Rn, recorded in Roztocze, are neither surprising nor particularly interesting, the maximum values from the Sudetes are remarkably high and significant. Any ^222^Rn activity concentration exceeding 3,000 Bq/dm^3^ should be regarded as very high. The value of 3,043 ± 6 Bq/dm^3^ measured in “Adit 19a” of a disused uranium mine in Kowary is currently the highest value of ^222^Rn activity concentration found in groundwaters in Poland. So high ^222^Rn activity concentrations in groundwaters of the Sudetes are caused by the raised level of parent ^226^Ra concentration in reservoir rocks, what was already stated by Przylibski and co-authors [7, 11, 22]. Such a high value of activity concentration of this isotope enables classifying these waters as high-radon waters, which can be hazardous to the health of the people who could be using them as drinking water, if it is not properly treated (de-radoned). At the moment, these waters are not utilized, though. Also, one could observe that according to radiation hormesis theory, such waters can be employed in balneotherapy. Putting aside the medical applications of high-radon waters, one should emphasize the fact that even the mean value of the ^222^Rn activity concentration measured by the authors in the Sudetes in the recent years is 541.4 Bq/dm^3^. It is a very high value, which confirms the opinion that radon waters are common in the Sudetes. This is consistent with the findings by Adamczyk-Lorenc [37], who found the value of the regional hydrogeochemical background of radon in Sudetic groundwaters to be 4–306 Bq/dm^3^. This confirms the necessity of monitoring ^222^Rn content in all those groundwater intakes in the Sudetes whose waters are to be consumed by people. In some of those intakes, de-radoning is necessary before the water can be fed into the water supply system. This complies with the recent World Health Organization recommendations [38] stipulating that ^222^Rn activity concentration in water in a public intake (before being fed into the water supply system) should be lower than 100 Bq/dm^3^. Unfortunately, such measurements are performed only occasionally now. Consequently, the Sudetes are an area where one could expect the common occurrence of radon waters and high-radon waters. These findings confirm the results of previous research by Przylibski and the co-authors [11].

Another significant issue is the high ^226^Ra activity concentration recorded in shallow circulation groundwaters in the Sudetes. While radon waters can be employed in balneotherapy and they can be used as drinking water, since removing radon from them is neither difficult nor expensive, waters containing increased ^226^Ra activity concentrations are a real problem. The values reaching 1.77 ± 0.11 Bq/dm^3^ found in shallow circulation infiltration waters are very high. Although they were recorded in the area of an exhausted uranium deposit in Kowary, similar values have also been occasionally recorded in other parts of the Sudetes, which is also confirmed by a high value of the arithmetic mean, 0.19 Bq/dm^3^ (for 70 locations; Table 1). This is also consistent with the earlier findings by Przylibski and the co-authors [16], and proves a non-zero probability that ^226^Ra ought to be removed from those groundwater intakes in the Sudetes whose groundwater is to be drunk by people. According to the recent recommendations from the World Health Organization [38], ^226^Ra activity concentration in drinking water should not exceed 1 Bq/dm^3^ (the so-called guidance level). This points to the necessity of determining also ^226^Ra activity concentration in all the Sudetic groundwater intakes whose water is to be consumed by people, and, if necessary, removing it from water or putting the intakes out of service.

In the other studied areas in southern Poland, i.e. the Carpathians and Roztocze, groundwaters do not contain such high concentrations of ^226^Ra or ^222^Rn. One can say that so far the values of ^222^Rn activity concentration recorded in groundwaters there have not posed any threat and they do not limit the use of these waters for human consumption. These are low-radon waters and radon-poor waters, i.e. containing from 10 to 99.9(9) and from 1 to 9.9(9) Bq/dm^3^ ^222^Rn respectively. Only in one spring ^222^Rn activity concentration reaches 104.2 ± 1.4 Bq/dm^3^. This concentration was determined in groundwater flowing thru granitoid rocks in High Tatra Range, a part of the Inner Carpathians. Consequently, most of examined groundwaters (with only one exception from High Tatra Range) may not be used in balneotherapy because of their ^222^Rn content. At the same time, the recorded maximum value (0.62 ± 0.04 Bq/dm^3^) and the arithmetic mean (0.51 Bq/dm^3^) of ^226^Ra activity concentration in shallow circulation groundwaters in four springs of the Carpathians are hardly any lower than the allowable values recommended by the WHO [38]. This means that it is also in the Carpathians where ^226^Ra activity concentration should be subject to routine monitoring in all the groundwater intakes whose water is to be drunk by people. Unfortunately, such determinations are not very common nowadays. Owing to a small number of data available for the Carpathians, continuing the started research seems to be essential for verifying its conclusions.

**Fig. 2:**
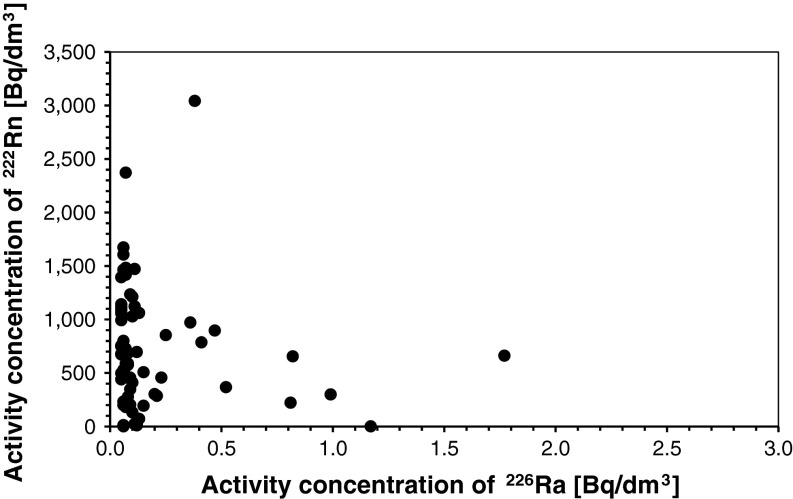
Correlation between ^222^Rn and ^226^Ra activity concentrations in shallow circulation groundwaters of the Sudetes

**Fig. 3:**
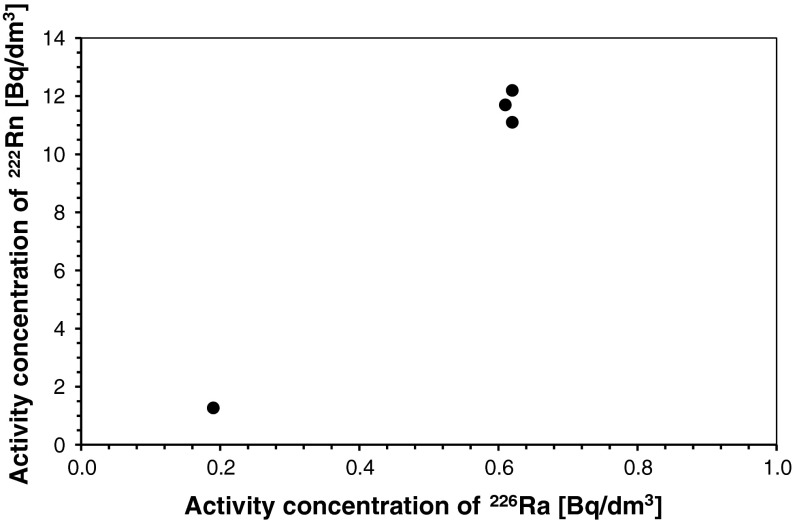
Correlation between ^222^Rn and ^226^Ra activity concentrations in shallow circulation groundwaters of the Carpathians

A comparison of data obtained from the Carpathians and the Sudetes in terms of ^226^Ra and ^222^Rn activity concentration demonstrates different characteristics of shallow circulation waters in these areas. The amounts and the ratios of ^222^Rn to ^226^Ra activity concentrations point to different genesis of radon dissolved in shallow circulation groundwaters in the Carpathians and in the Sudetes. In order to verify this observation for both areas, graphs showing the correlation between ^222^Rn activity concentration in groundwater and the activity concentration of ^226^Ra^2+^ dissolved in the same water have been drawn (Figs. 2, 3). The obtained correlations indicate that the activity concentration of ^222^Rn dissolved in groundwaters in the Carpathians is related to the concentration of the ion ^226^Ra^2+^ in these waters (for only four springs up to now). This is also confirmed by a high positive value of correlation coefficient between the activity concentrations of the studied isotopes in groundwaters in the Carpathians (cf. Table 1). Despite the high value of the correlation coefficient, the small number of available data makes it impossible to draw any ultimate conclusions from this correlation. Nevertheless, further research will determine if the observed regularity has an universal character for all the Carpathians.

Unlike in the Carpathians, one can ascertain that the activity concentration of ^222^Rn dissolved in shallow circulation groundwater in the Sudetes does not depend on the activity concentration of ions ^226^Ra^2+^ dissolved in the same water. This is clearly demonstrated by a small, much lower than 0.2, value of correlation coefficient (cf. Table 1). It shows that there is no statistically significant correlation between the activity concentrations of ^222^Rn and ^226^Ra in shallow circulation groundwaters in the Sudetes.

These findings demonstrate that in the Sudetes radon is dissolved in groundwater as gas released from reservoir rocks. In groundwater reservoir rocks, i.e. crystalline and, more rarely, sedimentary rocks, ^222^Rn is formed inside mineral crystals and grains or on their surface as a result of alpha decay of the parent ^226^Ra. This confirms earlier findings by Przylibski [7], who ascertained that ^226^Ra^2+^ ion decay in groundwaters is usually responsible for supplying <0.1 % of ^222^Rn dissolved in these waters. More rarely, this process supplies even 1 % of ^222^Rn dissolved in groundwaters, and only exceptionally these amounts are slightly higher than this value [7]. At the same time, a large number of ^222^Rn atoms dissolved in groundwaters in some of the Carpathians springs come from radioactive decay of ^226^Ra^2+^ ions dissolved in these waters. Thus, in the Carpathians, radon migrates to groundwaters both directly as gas from reservoir rocks and as a result of the decay of ^226^Ra^2+^ ions dissolved in these waters. Further research will enable determining the proportions of radon supplied into Carpathian shallow circulation groundwaters by both processes. So far, the results have demonstrated that these proportions will be different from those in the Sudetes. Unfortunately, the small amount of data gathered so far makes further research essential before ultimate conclusions can be formulated.

## Conclusions

The highest values of ^222^Rn and ^226^Ra activity concentration in shallow circulation groundwaters have been recorded in the Sudetes, where radon and high-radon waters have been found to occur. This is caused by the raised level of ^226^Ra concentration in reservoir rocks. In a groundwater outflow in “Adit 19a” of the disused uranium mine in Kowary, ^222^Rn activity concentration of 3,043 ± 6 Bq/dm^3^ was recorded, which is the highest value recorded in Poland so far. Such waters may be used in balneotherapy, in accordance with radiation hormesis theory. However, when being used as waters intended for human consumption, they should undergo the process of de-radoning in the intake, i.e. before being fed into the water-supply network. In the studied Sudetic groundwaters, high values of ^226^Ra activity concentration have been also found. This means that groundwaters tapped in the Sudetes with the aim of providing people with drinking water should be also monitored in terms of the concentration of this radionuclide. In extreme cases, when its content exceeds 1 Bq/dm^3^, radium should be removed from such waters or such intakes should be put out of service.

In the remaining part of the studied area in southern Poland, i.e. the Carpathians and Roztocze, no increased concentrations of ^222^Rn or ^226^Ra were recorded in shallow circulation groundwaters. There was found only one spring in the High Tatra Range, where radon water flows out, which could be used in balneotherapy there. Also, there is no need of removing radon from waters used as drinking water. In the Carpathians, however, shallow circulation groundwaters containing increased values of ^226^Ra activity concentrations may occur locally, although they do not exceed the allowable level for drinking waters recommended by the WHO. However, it is necessary to monitor the concentration of this isotope in waters intended for human consumption tapped from intakes in the Carpathians. The limited number of data gathered by the authors so far and the fact that the recorded values are significant in terms of radiological protection point to a necessity to continue the research in the Carpathians.

The authors have also observed that ^222^Rn dissolved in shallow circulation groundwaters in the Sudetes has different genesis to that in the Carpathians. In the Sudetes, the majority of radon dissolved in shallow circulation groundwaters is gas released from reservoir rocks. In the Carpathians, the amount of ^222^Rn dissolved in shallow circulation groundwaters is additionally influenced by the amount of radon formed as a result of the decay of the parent ^226^Ra^2+^ dissolved in these waters. Due to the small amount of data from the Carpathians, this conclusion must be verified by further research.

Owing to the incomplete recognition of the occurrence of ^222^Rn and ^226^Ra in shallow circulation groundwaters in southern Poland, the authors’ research will be continued. Because of the higher expected ^226^Ra content in deeper circulation waters and the related higher amount of total dissolved solids, the research will also comprise thermal waters, waters with high TDS and brines occurring in southern Poland and in the rest of the country.
